# Tackling obesity requires efficient government policies

**DOI:** 10.1186/2045-4015-1-18

**Published:** 2012-04-18

**Authors:** Michele Cecchini, Franco Sassi

**Affiliations:** 1 Health Division, OECD, 2 rue André Pascal, 75016 Paris, France

## Abstract

Changes in food supply and eating habits, combined with a dramatic fall in physical activity, have made obesity a global epidemic. Across OECD countries, one in two adults is currently overweight and one in six is obese. Children have not been spared, with up to one in three currently overweight. Obese people are more likely to develop diseases such as diabetes, cardiovascular disease, and cancer, and have a shorter life expectancy than people of normal weight. A prevention strategy combining health promotion campaigns, government regulation, counseling of individuals at risk in primary care, and paying special attention to the most vulnerable, would enhance population health at an affordable cost, with likely beneficial effects on health inequalities. Failure to implement such a strategy would impose heavy burdens on future generations. The new *IJHPR *paper by Ginsberg and Rosenberg illustrates how particular countries can assess alternative strategies for tackling obesity in a rigorous fashion.

This is a commentary on http://www.ijhpr.org/content/1/1/17/

## Commentary

Obesity is one of the main drivers of the global epidemic of chronic diseases. As such, it has risen to the top of the public health policy agenda worldwide. In the last 30 years obesity rates have doubled or tripled in many countries; currently, half of the OECD population qualifies as overweight. Although the prevalence of obesity in Israel is below the OECD average (14% vs. almost 17% in the OECD, according to the latest data [1]), rates are increasing as rapidly as in other countries. Women in disadvantaged socio-economic conditions are more likely than others to be or become obese, and children born to obese parents are three to four times more likely to be obese themselves than those born to normal-weight parents. Obesity has major impacts on labor productivity, income, and healthcare expenditures. Estimates suggest that up to 6% of healthcare budgets are spent on diseases related to obesity in the European region [2].

Economic analyses, like the one by Ginsberg and Rosenberg reported in this issue of the Journal [3], are badly needed to guide policy makers in tackling the rising tide of obesity. Especially in times of economic recession and severe public finance restrictions, governments ought to ensure greater priority is given to interventions that provide the largest health returns for the money spent. Ginsberg and Rosenberg conclude that a combination of prevention policies could increase population health by 32,671 QALYs (quality-adjusted life years) at a favorable cost-effectiveness ratio (US$12,800/QALY). These results are consistent with those of analyses jointly undertaken by OECD and WHO to assess the impacts of strategies to improve diets and increase physical activity [4]. The latter showed that the implementation and running costs of most prevention interventions are larger than the savings in healthcare expenditure generated by the same interventions. However, the extra costs are largely justified by the benefits generated, in terms of decreased morbidity and mortality from chronic diseases, which make these interventions a better investment than many treatments routinely provided by most health care systems.

Although interventions carried out in primary care settings, like some of those analyzed by Ginsberg and Rosenberg, appear to be effective and cost-effective, taking action only with high-risk individuals is not sufficient. These measures must be combined with community-level interventions aimed at shifting the entire BMI distribution and lowering the population mean. As there is no smoking gun responsible for obesity, there is no magic bullet to cure it. The current obesity epidemic is the result of a large number of changes that affected people's lifestyles over the past few decades, and it can only be tackled through a comprehensive prevention strategy approaching the problem from multiple angles, likely including regulatory and possibly fiscal measures.

Two key factors that may determine the success of community-based interventions are the ability of interventions to generate sustained changes in people's behaviors, and the extent to which individuals targeted by interventions respond. Ginsberg and Rosenberg have addressed the former factor in detail, by making alternative assumptions about the effects of interventions, but they have likely been too optimistic on coverage and uptake. Even the most comprehensive prevention program would struggle to reach a sizable proportion of the population and generate a change in health-related behaviors. In some cases appropriate measures can be taken to increase coverage and uptake, for instance financial incentives can be provided to increase the number of physicians who choose to participate in primary-care-based programs. But ensuring a meaningful uptake in community-based programs can be even more challenging.

OECD and WHO studies [5,6] provide evidence in support of the adoption of a multi-sectoral and multi-stakeholder approach. This conclusion is endorsed by the political declaration that resulted from the High-level Meeting of the UN General Assembly on NCDs, held in New York in September 2011 [7] in which it is recognized that "The incidence and impacts of non-communicable diseases can be largely prevented or reduced with an approach that incorporates evidence-based, affordable, cost-effective, population-wide and multisectoral interventions." A multi-sectoral approach, combining interventions that target different population groups, paying special attention to the most vulnerable, would enhance population health at an affordable cost, with likely beneficial effects on health inequalities.

## Competing interests

The authors declare that they have no competing interests.

## Authors' information

Franco Sassi, PhD, is a Senior Health Economist at the Organisation for Economic Cooperation and Development (OECD), based in Paris. Formerly a senior lecturer at the London School of Economics and Political Science, he held visiting positions in a number of leading North American universities. He has published extensively on the economics of public health and health care interventions, and he leads the OECD Economics of Prevention program.

Michele Cecchini, MD MSc, is a Health Policy Analyst at the Organisation for Economic Cooperation and Development (OECD), based in Paris. A public health physician by training, he is part of the OECD Economics of Prevention team and has played a major role in the analysis of policies to tackle obesity based on the OECD/WHO Chronic Disease Prevention (CDP) microsimulation model.
